# MicroRNA-125b modulates inflammatory chemokine CCL4 expression in immune cells and its reduction causes CCL4 increase with age

**DOI:** 10.1111/acel.12294

**Published:** 2015-01-23

**Authors:** Nai-Lin Cheng, Xiaochun Chen, Jiewan Kim, Alvin H Shi, Cuong Nguyen, Robert Wersto, Nan-ping Weng

**Affiliations:** 1 Laboratory of Molecular Biology & Immunology, National Institute on Aging, National Institutes of Health251 Bayview Blvd., Baltimore, MD, 21224, USA; 2 Flow Cytometry Unit, National Institute on Aging, National Institutes of Health251 Bayview Blvd., Baltimore, MD, 21224, USA

**Keywords:** aging, CCL4, immune cells, miR-125b, monocyte, and naïve CD8 T cell

## Abstract

Chemokines play a pivotal role in regulating the immune response through a tightly controlled expression. Elevated levels of inflammatory chemokines commonly occur with aging but the mechanism underlying this age-associated change is not fully understood. Here, we report the role of microRNA-125b (miR-125b) in regulating inflammatory CC chemokine 4 (CCL4) expression in human immune cells and its altered expression with aging. We first analyzed the mRNA level of CCL4 in eight different types of immune cells including CD4 and CD8 T-cell subsets (naïve, central and effector memory), B cells and monocytes in blood from both young (≤42 years) and old (≥70 years) adults. We observed that monocytes and naïve CD8 T cells expressed higher levels of CCL4 and exhibited an age-related increase in CCL4. We then found the level of miR-125b was inversely correlated with the level of CCL4 in these cells, and the level of miR-125b was reduced in monocytes and naïve CD8 T cells of the old compared to the young adults. Knock-down of miR-125b by shRNA in monocytes and naïve CD8 T cells led to an increase of CCL4 protein, whereas enhanced miR-125b expression by transfection in naïve CD8 T cells resulted in a reduction of the CCL4 mRNA and protein in response to stimulation. Finally, we demonstrated that miR-125b action requires the ‘seed’ sequence in 3′UTR of CCL4. Together these findings demonstrated that miR-125b is a negative regulator of CCL4 and its reduction is partially responsible for the age-related increase of CCL4.

## Introduction

Chemokines are small cytokines with an array of fundamental immune functions. One of the prominent roles of chemokines is directing lymphocyte migration from the bloodstream to lymphoid organs or to sites of inflammation (Nibbs & Graham, 2013). Within lymphoid organs or tissues, chemokines also direct the encounter between antigen-presenting dendritic cells (DCs) and antigen-specific T cells to ensure T-cell priming and subsequent activation (Castellino *et al*., 2006; Matloubian & Cyster, 2012). The effects of chemokines are associated with their level of expression (Anderson, 2008; Sung *et al*., 2012) and the expression of their receptors on target lymphocytes and macrophages (Allen *et al*., 2007). The numerous members of chemokine families and their receptors offer an unparalleled complexity in the regulation of chemokine functions. Given their critical role in immune function, the expression of chemokines and their receptors are tightly regulated.

The decline of immune function with age is characterized by a reduced adaptive immune response and increased systemic chronic inflammation (Weng, 2006; Goronzy & Weyand, 2013; Shaw *et al*., 2013). A hallmark of age-associated chronic inflammation without an overt infection is the elevated levels of inflammatory biomarkers such as C-reactive proteins, interleukin-6 (IL-6), chemokines CCL2, CCL3, and CCL4 in blood (Chiu *et al*., 2006; Seidler *et al*., 2010; Franceschi & Campisi, 2014; Wang & Casolaro, 2014). Although the precise etiology of age-associated chronic inflammation is not fully understood, several factors have been implicated including (i) damaged macromolecules and cells that activate the innate immune system (Dall'Olio *et al*., 2013); (ii) persistent or reactivation of latent viral infections (Wikby *et al*., 2006), and (iii) cellular senescence (Salama *et al*., 2014). Elevated IL-6 and other factors are strong predictors of age-related morbidity and mortality (Varadhan *et al*., 2014) but the precise role of these factors in various age-related diseases in humans remains to be elucidated.

Inflammatory chemokine (CC motif) ligand 4 (CCL4, aka MIP-1β) is produced by a wide variety of cells including: immune cells (monocytes, B and T cells), fibroblasts, endothelial, and epithelial cells (Maurer & von, 2004). CCL4 binding to its major receptors, the G protein-coupled receptors CCR5 and CCR8, initiates the migration of immune cells (e.g., monocytes, nature killer cells, and T cells), dendritic cell maturation, activation of granulocytes and T cells, and T cell differentiation (Sallusto *et al*., 1999; Tang & Cyster, 1999; Castellino *et al*., 2006). Due to its broad role in immune function, the expression of CCL4 is tightly regulated during T-cell differentiation and activation (Cristillo *et al*., 2003). Increased expression of CCL4 was reported in the monocytes and blood of elderly individuals (Chiu *et al*., 2006; Seidler *et al*., 2010) as one of the age associated elevated inflammatory cytokines. Although its precise contribution to the decline of immune function and increase of diseases with aging require further study, CCL4 may have an impact on a broad range of immune cell functions including migration, growth, and differentiation that are altered during aging.

MicroRNAs (miRNAs) are noncoding transcripts of ∽22 nucleotides that inhibit translation and/or induce the degradation of target mRNAs (Baek *et al*., 2008; Selbach *et al*., 2008). Over 2500 miRNAs have been identified in the human (miRbase release 21, http://www.mirbase.org) and may regulate over 60% of gene transcripts (Friedman *et al*., 2009). In the immune system, several miRNAs have been identified that modulate immune cell recruitment (Yang *et al*., 2012), development and differentiation by modulating the expression of specific target genes (Li *et al*., 2007; Xiao *et al*., 2007; Zhou *et al*., 2008). miR-125b is involved in the fate decision of hematopoietic stem cells (HSC), as high amounts of miR-125b tend to lead HSC development toward to the lymphoid lineage (Ooi *et al*., 2010). In T cells, enhanced miR-125b expression keeps CD4 T cells in the naïve stage from differentiating (Rossi *et al*., 2011); miR-125b also modulates expression of ETS1 and STAT3 in T cells (Luo *et al*., 2013). In the myeloid lineage, miR-125b represses interferon regulatory factor 4 (IRF4) (Chaudhuri *et al*., 2011) and regulates inflammation by downregulating its direct target, TNFα (Tili *et al*., 2007), in macrophages. Finally, miR-125b is involved in several diseases including myelodysplastic syndrome, leukemia (Bousquet *et al*., 2008, 2010) and the pathogenesis of Alzheimer's disease (Banzhaf-Strathmann *et al*., 2014); interestingly, it is reported that CCL4 expression is altered in patients with Alzheimer's disease (Xia & Hyman, 1999), although the relationship between miR-125b and CCL4 is not known. Together, these studies have demonstrated that miR-125b has a broad role in the regulation of immune cell function including both lymphoid and myeloid lineages. However, whether miR-125b is involved in the age-associated change of immune function is not known.

## Results

### Increased CCL4 and decreased miR-125b expressions in monocytes and naïve CD8 T cells with age

**Figure 1:**
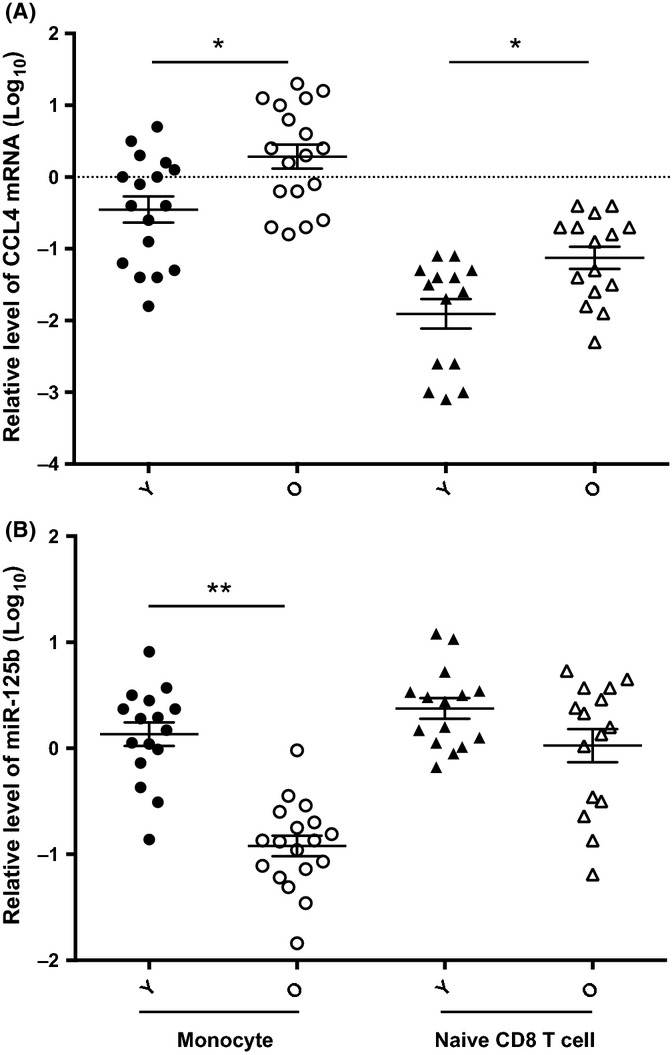
Inverse correlation of CCL4 and miR-125b expression in human monocytes and CD8 naïve T cells. (A) Relative CCL4 mRNA level in monocytes and naïve CD8 T cells in young and old adults. Monocytes (Young ≤ 42 years, *N* = 16 and Old ≥ 70 years, *N* = 18), naïve CD8 T cells (Young = 15 and Old = 15), were isolated from blood of young and old adults by immunomagnetic separation and cell sort, and CCL4 mRNA level were measured by RT-qPCR and first normalized to ACOX-1 and then normalized to a standard generated from PBMCs of five normal subjects (Log_10_). (B) Relative miR-125b level in monocytes (Young = 16 and Old = 18) and naïve CD8 T cells (Young = 15 and Old = 15) in young and old adults. miR-125b level was measured by RT-qPCR and first normalized to RNU6B and then normalized to a standard generated from PBMCs of five normal subjects (Log_10_). Multifactor ANOVA with Benjamini–Hochberg procedure was used, and ** and *P < 0.01 and <0.05, respectively.

Our previous study observed an elevated level of inflammatory chemokine CCL-4 in the blood from old individuals (Chiu *et al*., 2006). To determine whether the age-related increase of CCL4 in the blood is due to an increased expression of CCL4 in some or most types of immune cells in the blood, we compared the levels of CCL4 in eight types of immune cells (monocytes, B cells, and naïve and memory subsets of both CD4 and CD8 T cells) from old (age ≥ 70 years) and young (age ≤ 42 years) adults. We found that monocytes expressed the highest level of CCL4 mRNA, followed by CD8 memory subsets (Fig.1A and Fig. S1A). Compared between young and old adults, significant increases of CCL4 mRNA were observed in monocytes (5.4-fold, *P *<* *0.05) and naïve CD8 T cells (5.0-fold, *P *<* *0.05) (Fig.1A) but not in the other six types of immune cells tested (Fig. S1A). These findings illustrate not all types of immune cells alter expression of CCL4 comparably with age; in fact, monocytes are the major source of elevated CCL4 expression seen with age.

To examine the potential cause of this age-associated increase of CCL4 expression, we focused on the role of miR-125b as it has a binding site at the 3′UTR region of CCL4 from computational analysis. First, we measured the level of miR-125b in the same eight types of immune cells and found an overall inverse correlation between miR-125b and CCL4 in both young and old humans (*R*^2^ = 0.38 and 0.54 for young and old, respectively) (Fig. S2). Among these immune cell types, monocytes expressed the highest level of CCL4, whereas naïve CD4 and CD8 T cells expressed the highest levels of miR-125b. Compared to old adults, young adults had significantly higher level of miR-125b in monocytes (11.3-fold, *P *<* *0.01) and higher, but not statistically significant, in naïve CD8 T cells (2.2-fold, *P *=* *0.18); there was no obvious difference in the six types of cells (Fig.1B and Fig. S1B).

### An inverse correlation of CCL4 and miR-125b expression in monocytes

**Figure 2:**
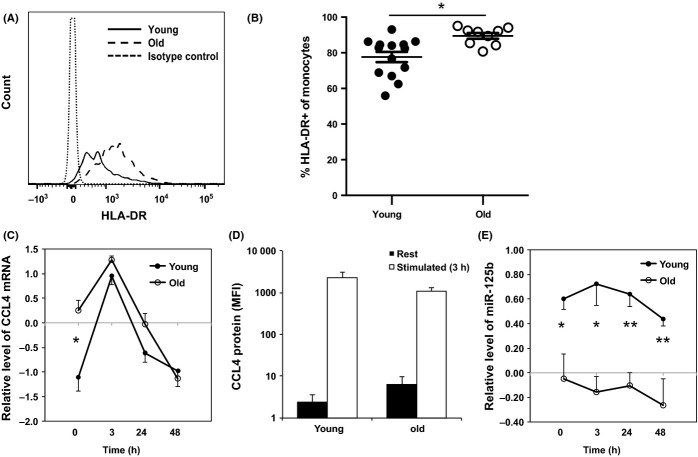
Activation-induced change of CCL4 and miR-125b in monocytes from young and old adults. (A) A representative histogram of HLA-DR staining on monocytes from young and old adult. The positive staining of HLA-DR was based on the isotype control staining. (B) Percentages of HLA-DR^+^ monocytes in young (*N* = 14) and old (*N* = 9) adults. Whole blood samples were stained with antibodies against CD14, HLA-DR, and other markers, analyzed by FACSCaton II, and percentages of HLA-DR^+^ in monocytes were presented. (C) Relative levels of CCL4 mRNA in monocytes after *in vitro* stimulation. The relative levels (in Log_10_) were after normalization to a standard generated from PBMCs of five normal subjects. (D) Intracellular CCL4 protein staining of freshly isolated (rest) and LPS stimulated (3 h) monocytes. The average mean fluorescent intensity (MFI) is shown (young = 6 and old = 11). (E) Relative levels of miR-125b in monocytes after *in vitro* stimulation. Freshly isolated monocytes from young (*N* = 6) and old (*N* = 6) adults were stimulated with LPS for 3, 24, and 48 h. The levels of CCL4 mRNA and miR-125b were determined by RT-qPCR and normalized to ACOX-1 and to RNU6B first and then normalized to a standard generated from PBMCs of five normal subjects in Log_10_ scale, respectively. Student's t-test was used for the analysis, **P < 0.01, and *P < 0.05 used in this and following figures.

To determine whether the elevated level of CCL4 mRNA in the monocytes of old subjects was related to activation, we compared a panel of monocyte activation markers including, CD69, PD-1, and HLA-DR (Barbosa *et al*., 2012) between young and old subjects. We found a significant increase in HLA-DR^+^ monocytes from an average of 78% in young adults to an average of 90% in the old (Fig.2), implying that activation may contribute to an enhanced expression of CCL4 in monocytes with aging. To directly examine the activation effect on monocytes, we stimulated freshly isolated monocytes with LPS and measured CCL4 mRNA and protein, and miR-125b levels before and after stimulation. We observed the CCL4 mRNA level increased at 3 h and reduced at 24 and 48 h after LPS stimulation in monocytes from both young and old adults by quantitative RT–PCR (Fig.2C). We then used the flow cytometry method to measure CCL4 protein. Before stimulation, CCL4 protein level was very low to undetectable in monocytes of most study subjects except a few old subjects. However, LPS stimulation significantly increased CCL4 protein level in monocytes of both old and young adults (Fig.2D).

**Figure 3:**
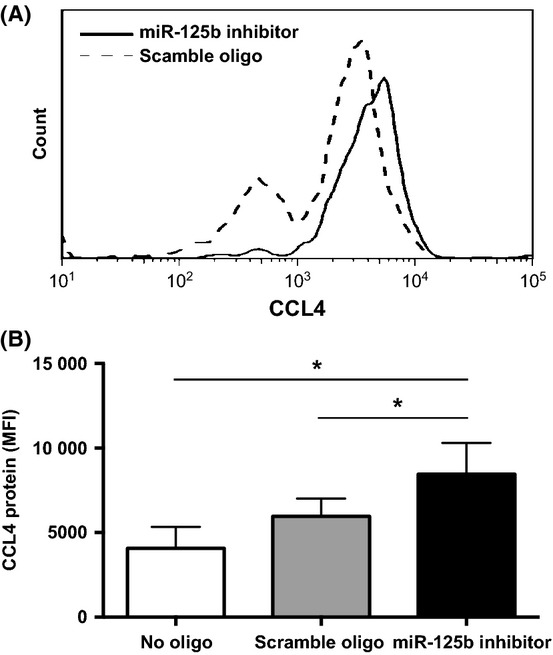
Reduction of miR-125b levels in monocytes resulted in increased CCL4 protein expression. (A) A representative graph of CCL4 staining in monocytes transfected with hsa-miR-125b miRCURY™ LNA inhibitor or a scrambled oligonucleotide. (B) Level of CCL4 protein (MFI) in monocytes transfected with hsa-miR-125b miRCURY™ LNA inhibitor or a scrambled oligonucleotide or no oligo. Freshly isolated monocytes were transfected with either hsa-miR-125b miRCURY™ LNA inhibitor (*N* = 13), or a scrambled oligonucleotide (*N* = 13), or no oligo (*N* = 6) for 16 h and followed by LPS stimulation for 3 h. Cells were harvested, and the levels of intracellular CCL4 protein were measured by the mean fluorescence intensity under the gate of positively transfected cells based on the oligonucleotide-linked fluorescein or no gate for no oligo transfection control.

In parallel, we examined the level of miR-125b and found it was significantly lower in the monocytes from old adults than from young adults (Fig.2E). The difference of the miR-125b level was significant at all four time points before and after LPS stimulation between young and old adults. To determine whether the reduction of miR-125b was responsible for the elevated expression of CCL4 in monocytes, we transfected a miR-125b-specific inhibitor (hsa-miR-125b miRCURY™ LNA inhibitor) into freshly isolated monocytes from young adults. We found a significant increase of CCL4 protein in monocytes transfected with the miR-125b-specific inhibitor after LPS stimulation compared to the controls monocytes transfected with either the scramble-miR or no miR) (*P *<* *0.05) (Fig.3). These findings suggested the reduction of miR-125b is at least partially responsible for the increase of CCL4 in monocytes and may contribute to the age-related increase of CCL4 in monocytes.

### An inverse correlation of CCL4 and miR-125b in resting and activated naïve CD8 T cells

**Figure 4:**
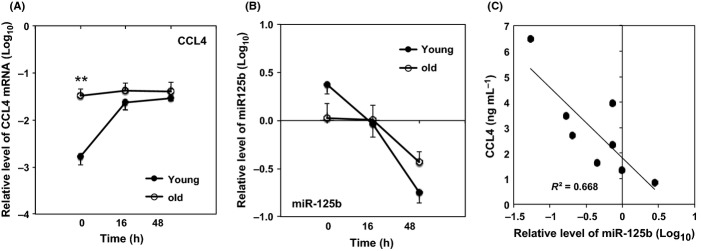
Activation-induced change of CCL4 and miR-125b in naïve CD8 T cells before and after stimulation from young and old adults. (A) Relative CCL4 mRNA and (B) Relative miR-125b levels in naïve CD8 T cells. (C) Correlation of CCL4 protein and miR-125b (*N* = 8). R^2^ = 0.7 (linear regression analysis); *P *=* *0.01. Freshly isolated naïve CD8 T cells from young (*N* = 13–16) and Old (*N* = 14–16) were stimulated *in vitro* by antibodies against CD3 and CD28. Cells were harvested at the indicated time for analyses of CCL4 mRNA and miR-125b, and culture supernatant was collected for measurement of CCL4 protein. The levels of CCL4 mRNA and miR-125b were measured by quantitative RT-PCR and normalized to ACOX1 and RNU6B first and then normalized to a standard generated from PBMCs of five normal subjects in Log_10_ scale, respectively. Both CCL4 and miR-125b were significantly different between 0 and 16 h, and between 0 and 48 h (*P *<* *0.05). CCL4 protein in culture supernatant was determined by ELISA.

As naïve T cells expressed the highest level of miR-125b among the eight types of immune cells from peripheral blood, we sought to determine how the expression of CCL4 and miR-125b in naïve CD8 T cells changed after stimulation with antibodies against CD3 and CD28 (anti-CD3/CD28) *in vitro*, and whether the expression of CCL4 and miR-125b was altered between young and old adults after stimulation. Naïve CD8 T cells from young adults expressed significantly higher levels of CCL4 mRNA at 16 and 48 h (*P *<* *0.01) and significantly reduced levels of miR-125b at both 16 and 48 h (*P *<* *0.01) compared to the resting state (Fig.4A,B). In contrast, naïve CD8 T cells from old adults showed an increase of CCL4 mRNA after activation but did not reach statistical significance, as well as a reduction of miR-125b after activation which was only significant at 48 h (*P *<* *0.05). Comparing between young and old adults, the differences of CCL4 and miR-125b were more prominent before stimulation and dissipated after stimulation (Fig.4A,B). We then extended the measurement of CCL4 mRNA to protein in the culture supernatant of naïve CD8 T cells after stimulation and found that the levels of the CCL4 protein and miR-125b were also inversely correlated (*R*^2^ = 0.7) (Fig.4C).

### Modulation of miR-125b expression resulting in an inverse change of CCL4 in naïve CD8 T cells

**Figure 5:**
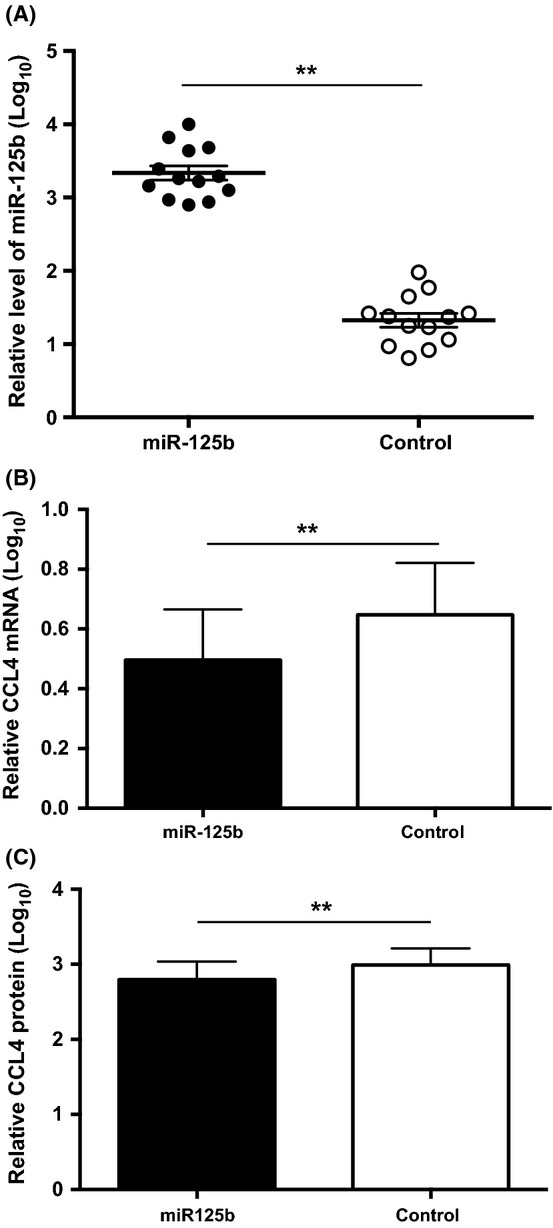
Enhanced miR-125b expression and reduced CCL4 expression in naïve CD8 T cells. (A) Enhanced miR-125b level (∽78-fold) in naïve CD8 T cells transfected with GFP-tagged miR-125b expressing vector compared to naïve CD8 T cells transfected with GFP control vector. Twelve hours post-transfection, GFP-positive cells were isolated by cell sorting for determining the levels of miR-125b. (B) Reduced expression of CCL4 mRNA (*N* = 9) and (C) protein (*N* = 12) in miR-125b enriched naïve CD8 T cells as compared to control cells. Supernatants were collected after 16 h stimulation with anti-CD3 and anti-CD28 from transfected cells. The data were log transformed and presented as mean and SEM. Paired Student t-test was used (** *P *<* *0.01). The concentration of CCL4 protein ranges from 0.2 to 12 ng/mL.

To further test whether miR-125b modulates CCL4 expression in naïve CD8 T cells, we cloned miR-125b in an expression vector expressing GFP and transfected it to freshly isolated naïve CD8 T cells. Transfected naïve CD8 T cells were enriched based on GFP by cell sorting and stimulated with anti-CD3/CD28 for 16 h. The levels of miR-125b were significantly higher in miR-125b transfected cells than in the control vector transfected cells (Fig.5A), whereas the CCL4 mRNA (Fig.5B) and protein (Fig.5C) were significantly reduced in the miR-125b-transfected than the control vector transfected naïve CD8 T cells. We also transfected a miR-125b-specific inhibitor (hsa-miR-125b miRCURY™ LNA inhibitor) into freshly isolated naïve CD8 T cells and found an increase of CCL4 protein (data not shown). These findings suggest that miR-125b negatively regulates CCL4 expression in naïve CD8 T cells.

### Expression of pri-miR-125bs in monocytes and naïve CD8 T cells with age

**Figure 6:**
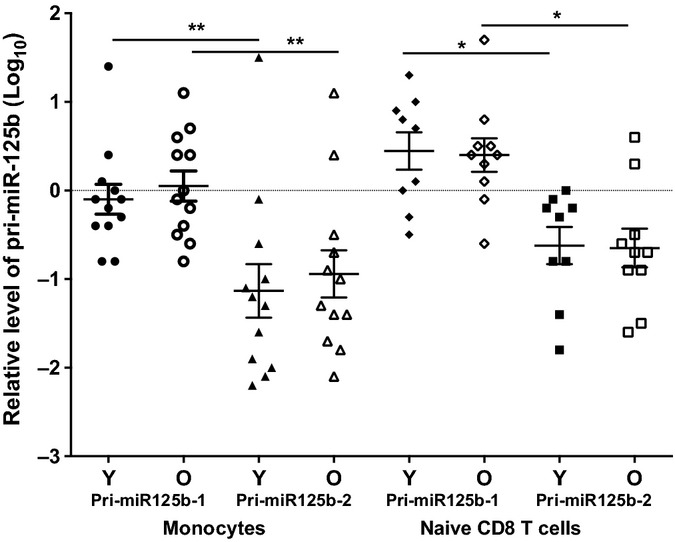
pri-miR-125b-1 and pri-miR-125b-2 in monocytes and naïve CD8 T cells from young and old adults. Monocytes (Young ≤ 42 years, *N* = 12 and Old ≥ 70 years, *N* = 12) and naïve CD8 T cells (Young = 9 and Old = 10) were isolated from the blood of normal young and old donors. Relative levels of pri-miR-125b-1 and pri-miR-125b-2 were first normalized to ACOX1 and then normalized to a standard generated from the PBMCs of five normal subjects in Log_10_ scale.

To determine the potential cause of the change in miR-125b level in the old humans, we further evaluated the levels of primary miR-125b transcripts. The mature miR-125b is generated from two miR-125b genes: miR-125b-1 and miR-125b-2, which are located on chromosome 11 and 21, respectively. We examined both pri-miR-125b-1 and pri-miR-125b-2 by the quantitative RT–PCR method and found that pri-miR-125b-1 was significantly more abundant than pri-miR-125b-2 in monocytes (*P *<* *0.01) and naïve CD8 T cells (*P *<* *0.05) (Fig.6). However, the levels of pri-miR-125bs (pri-miR-125b-1 + pri-miR-125b-2) in monocytes and naïve CD8 T cells were not significantly different between the young and old adults (Fig.6). This indicates that the reduction of mature miR-125b in the monocytes of old adults was not due to the reduced transcription of pri-miR-125bs.

### Inhibition of CCL4 by miR-125b through a specific site at the 3′UTR of CCL4

**Figure 7:**
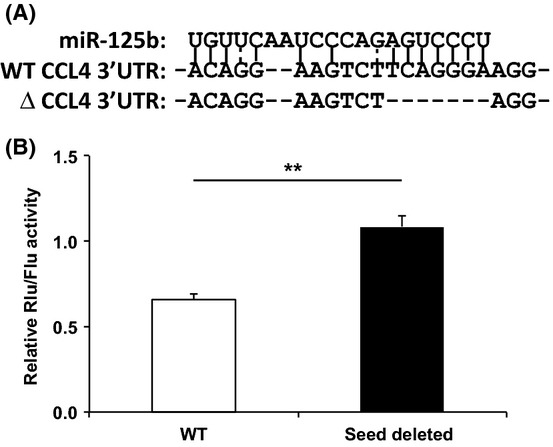
Inhibition of CCL4 expression by miR-125b through a binding site at the 3′UTR of CCL4. (A) The diagram of the putative miR-125b binding site at the 3′UTR of CCL4 and miR-125b binding site deleted mutant. The complementary base pairing between CCL4 3′UTR and miR-125b is predicted by ‘MicroRNA.org’. (B) Inhibition of CCL4 by miR-125b requiring the specific sequence at the 3′UTR of CCL4. miR-125b overexpressed Jurkat cells were further transfected with a renilla luciferase reporter containing either wild-type 3′UTR or miR-125b binding site deleted 3′UTR of CCL4. The renilla luciferase activity was normalized to the constitutively expressed firefly luciferases. The data are presented as the means of three independent experiments and SEM (*N* = 9, **P <* *0.01).

To determine whether miR-125b directly modulates CCL4 expression, we deleted the putative miR-125b binding site (‘seed’) at the 3′UTR region of CCL4 mRNA by constructed a ‘seed deleted’ and wild-type (WT) 3′UTR of CCL4 in dual luciferase reporter vectors (Fig.7A) and established a miR-125b enriched Jurkat cell line. Transfecting the WT or ‘seed deleted’ CCL4 3′UTR-containing reporter to the miR-125b enriched Jurkat cells showed significantly lower (*P *<* *0.001) activity of the luciferase reporter from the transfected cells with WT 3′UTR construct compared to the ‘seed deleted’ mutant (Fig.7B). This result confirmed that the inhibitory effect of miR-125b on CCL4 expression requires the miR-125b target sequence at the 3′UTR of CCL4.

## Discussion

Understanding how chemokine expression is regulated in immune cells and what factors influence their altered expression with aging are critical to elucidating the precise functions of chemokines in an immune response and the mechanisms of its alteration with aging. In this report, we showed that miR-125b expression is inversely correlated with CCL4 mRNA level in immune cells in peripheral blood. Among them, monocytes and naïve CD8 T cells exhibited an age-related change with increased CCL4 mRNA and reduced miR-125b. Modulation of the level of miR-125b with either reduction (monocytes and naïve CD8 T cells) or enhancement (naïve CD8 T cells) led to a reciprocal change of the CCL4 protein in monocytes and naïve CD8 T cells. The age-associated reduction of miR-125b appeared to not be related to the transcription of primary miR125b-1 or miR125b-2, but may be related to the miR-125b maturation process. Finally, we demonstrated that the action of miR-125b on CCL4 requires a specific sequence at the 3′UTR of CCL4 mRNA. These findings suggest the expression of CCL4 is modulated by miR-125b and alterations in the balance between CCL4 and miR-125b are responsible for the age-associated increase of the inflammatory chemokine, CCL4.

miR-125b has a broad spectrum of functions in immune cells including, regulating the output of hematopoietic lineage cells (O'Connell *et al*., 2010; Ooi *et al*., 2010), inhibiting B-cell differentiation to plasma cells via inhibition of B lymphocyte-induced maturation protein-1 (BLIMP-1) in the germinal center (Gururajan *et al*., 2010), and inhibiting expression of TNFα in macrophages (Tili *et al*., 2007). Our finding suggests that reduced miR-125b levels in monocytes contribute to the increase of CCL4 in the blood in the old donors. Whether miR-125b also plays a role in the age-related increase of myeloid lineage cells such as monocytes requires further study. Our finding of the inhibitory role of miR-125b in CCL4 expression expands the role of miR-125b in lymphocyte migration, inflammation, and aging. While high levels of miR-125b in monocytes and resting naïve CD8 T cells may prevent the translation of unwanted CCL4 transcript, a decreased miR-125b expression is needed to enhance activation-induced CCL4 expression. This fine-tuning of the level of CCL4 by miR-125b ensures CCL4 is properly expressed in monocytes and naïve CD8 T cells at rest and after activation. Like many of the inflammatory cytokines that increase with age, the precise consequences of the dysregulation of CCL4 on the functions of monocytes and naïve CD8 T cells, such as inflammatory states or altered migrations, remain to be elucidated.

Elevated levels of inflammatory cytokines and chemokines are one of the hallmarks of an aging immune system (Seidler *et al*., 2010; Franceschi & Campisi, 2014; Wang & Casolaro, 2014). Although such changes have been linked to increased morbidity and mortality of older individuals, the mechanism underlying these changes in gene expression is not completely understood. Here, we observed a decrease of miR-125b associated with a significant increase of CCL4 mRNA in monocytes, the major producer of CCL4, in old adults compared to young adults. We also observed a reduction of miR-125b in monocytes leads to enhanced expression of the CCL4 protein in response to LPS stimulation; thus, alteration of the balance between CCL4 and miR-125b may be one of the mechanisms underlying the age-associated increase of CCL4. Furthermore, monocytes from old subjects appear to consist of more activated cells than monocytes from young subjects, providing another possible mechanism of age-associated increase of CCL4. However, the precise consequences on immune function that occur as a result of the reduction of miR-125b/increase of CCL4 observed in the monocytes of old adults remain to be determined.

The level of mature miR-125b is potentially regulated at different stages such as transcription, assembly, and stability. It is reported that NFκB upregulates miR-125b expression (Tan *et al*., 2012) and is increased in the nucleus with aging (Donato *et al*., 2008); however, our data show no obvious alteration in pri-miR-125b transcription in monocytes and naïve CD8 T cells with aging. Thus, the reduction of mature miR-125b with age could be due to the alteration of miR-125b maturation. Primary miRNA processing appears to be by miRNA-specific mechanisms (Winter *et al*., 2009). Several nuclear factors are implicated in the process including FUS/TLS (fused in sarcoma/translocated in liposarcoma) which facilitate Drosha recruitment to certain specific miRNAs (e.g., miR-125b) in a neuroblastoma cell line (Morlando *et al*., 2012). Whether advancing age impairs pri-miR-125b processing remains to be determined. Further characterization of the target cytokines of miR-125b and elucidation of the biosynthesis of miR-125b in immune cells is necessary to fully understand the scope and mechanism of miR-125b function in T-cell differentiation and immune cell aging. In view of its broad function, miR-125b could serve as a potential target for clinical applications in fine-tuning the cytokine/chemokine levels in disease conditions and aging.

## Experimental procedures

### Study subjects

A total of 106 human adults who are either healthy blood donors (*N* = 59, <70 years old) or old participants (*N* = 47, ≥70 years old) of the Baltimore Longitudinal Study of Aging (BLSA) (Table S1) were included in the study. Blood collection was under Institutional Review Board-approved protocols.

### Reagents

Antibodies against CD3 and CD28-coupled magnetic beads (anti-CD3/28), penicillin, and streptomycin were from Invitrogen (Grand Island, NY, USA). Anti-CD62L-FITC, anti-CD45RA-TC, anti-CD14-Pacific Blue, anti-PD-1-APC, anti-HLA-DR-Alexa Fluor® 700, CD69-PE, and CD16-FITC were from Biolegend (San Diego, CA, USA). Anti-human CCL4-PerCP and its mouse IgG2B-PerCP isotype control were from R&D systems (Minneapolis, MN, USA). *HindIII*,*BamHI*,*XhoI*,*NotI*, and *BstEII* were from New England BioLabs (Ipswich, MA, USA). Puromycin and other chemicals were purchased from Sigma-Aldrich (St. Louis, MO, USA).

### Isolation of human immune cells from blood and stimulation of monocytes and naïve CD8 T cells *in vitro*

Peripheral blood was collected at the clinic of the National Institute on Aging (NIA, Baltimore, MD, USA). Mononuclear cells were isolated by Ficoll (GE Healthcare, Pittsburgh, PA, USA) gradient centrifugation. Following manufacture's instruction, subsets of immune cells were isolated as monocytes (CD14^+^CD16^−^) by a Monocyte Isolation Kit II (Miltenyi, San Diego, CA, USA) and B cells by Dynabeads® CD19 pan B (Life Technology, Grand Island, NY, USA). The procedure of isolation of naive and memory T cells was described previously (Araki *et al*., 2009). Naïve (CD62L^+^CD45RA^+^), central memory cells (CD62L^+^CD45RA^−^), and effector memory cells (CD62L^−^CD45RA^−^) of both CD4 and CD8 T cells were further isolated by a cell sorter (MoFlo, Dako Cytomation, Carpinteria, CA, USA). The purity of sorted naive and memory T cells was > 96%.

Freshly isolated monocytes and naïve CD8 T cells were stimulated with lipopolysaccharide (LPS, 100 ng/mL) and with anti-CD3/28-coupled beads at a cell:bead ratio of 1:1 in RPMI 1640 with 10% FBS and penicillin (10 U/mL)/streptomycin (10 μg/mL), respectively. Stimulated cells were harvested at the indicated time for analyses of CCL4 mRNA and/or protein, and miR-125b and pri-miR-125b-1 and pri-miR-125b-2.

### Quantitative RT–PCR of mRNA, pri-miRNA, and miRNA

The procedure of the quantitative RT–PCR was described previously (Araki *et al*., 2009). Briefly, cDNA was synthesized with oligo-dT plus random hexamers (Invitrogen). Primer sequences for CCL4 are as follows: forward (5′-AAGCTCTGCGTGACTGTCCTGT-3′) and backward (5′-AAGCTTCCTCGCGGTGTAAGA-3′). The mRNA levels of CCL4 were determined by quantitative RT–PCR using 2x SYBR Green PCR Master Mix (Applied Biosystems, Grand Island, NY, USA) and normalized to a lymphocyte housekeeping gene, acyl-Coenzyme A oxidase 1 (ACOX1), as described previously (Araki *et al*., 2009). Quantitative RT–PCR of mature miRNAs (miR-125b and RNU6B) and pri-miRNAs (hsa-miR-125b-1 and hsa-miR-125b-2) was performed using the TaqMan® MicroRNA Assays with the primer sets form Applied Biosystems following the manufacturer's instruction. The value of miR-125b threshold cycle (Ct) was normalized to RNU6B, and the value of pri-miRNAs was normalized to ACOX1. PCR was performed on 7900HT Fast Real-Time PCR System (Applied Biosystems). The relative levels of CCL4/ACOX1, miR-125b/RNU6B, and pri-miRNAs/ACOX1 were presented in Log_10_ scale, respectively. To compare the relative levels of gene expression, we established a standard of these genes (CCL4, miR-125b, and pri-miR-12bs) using PBMCs from five normal adults and then normalizing the results of each sample to the standard.

### Analysis of monocyte activation markers by flow cytometry

Cell surface staining was performed on whole blood samples in EDTA, and the whole procedure was conducted under room temperature. One hundred microlitre of whole blood was blocked with 5 μL of Fc blocker (Human TruStain FcX™; Biolegend) for 5 min before incubating 20 min at dark with antibody cocktail, including anti-CD14-Pacific Blue, anti-PD-1-APC, anti-HLA-DR-Alexa Fluor® 700, and CD69-PE. After erythrocyte lysis, sample was analyzed using a FACSCanto™ II (Becton Dickinson, San Diego, CA, USA). Ten thousand events were acquired. Negative controls were stained with isotype-matched monoclonal antibodies (Biolegend). The results are analyzed under the gated monocyte (CD14^+^) population and expressed as the percentages of HLA-DR^+^, PD-1^+^, and CD69^+^.

### Inhibiting miRNA in human monocytes and naïve CD8 T cells

Four million of the freshly purified human monocytes/naïve CD8 T cells were electroporated either with or without 1.5 μm of hsa-miR-125b miRCURY™ LNA inhibitor (EXIQON, Woburn, MA, USA) or Scramble-miR inhibitor Control (Negative control A, EXIQON). The electroporation was carried out using a P3 Primary Cell 96-well Nucleofector™ Kit (Lonza) following the manufacturer's instructions. Next day (16 h after transfection), monocytes were stimulated for 3 h with LPS (100 ng/mL) plus GolgiPlug™ (1 μL/mL; BD Biosciences, San Jose, CA, USA), and naïve CD8 T cells were stimulated by anti-CD3/CD28 antibodies for at 3 h in the presence of GolgiPlug. The levels of CCL4 in electroporated cells were determined by intracellular staining with anti-CCL4 (R&D systems) as described below.

### Intracellular chemokine analysis by flow cytometry

Cells were fixed with 100 μL of FOXP3 Fix/Perm Buffer (BioLegend) over night at 4°C. The fixed and permeabilized cells were stained with mouse anti-human CCL-4/MIP-1 beta PerCP conjugated monoclonal antibody or mouse IgG2B-PerCP isotype control (R&D Systems). All samples were acquired and analyzed on BD Accuri™ C6 Flow Cytometer (BD Biosciences).

### Overexpressing miR-125b in primary human naïve CD8 T cells

A 248-bp hsa-mir-125b-1 containing fragment was amplified from human genomic DNA with the following primers: forward (5′-cccggatccTCTTAGTTATGAACCTCG-3′) and backward (5′-cccaagcttTACTCAATCACCTCAGAC-3′). The PCR product was digested with *HindIII* and *BamHI*, cloned into the pSilencer 4.1-CMV puro vector (Ambion, Grand Island, NY, USA), and confirmed by sequencing. The miR-125b expression vector and control empty vector were digested with *NotI* to insert GFP gene for sorting purpose. 0.4 μg/million cell of GFP plus miR-125b vector or control GFP vector was transfected into freshly isolated naïve CD8 T cells using Human T cell Nucleofector kit (Lonza, Allendale, NJ, USA). GFP-positive cells were isolated by a cell sorter (MoFlo) at ∽12 h post-transfection; this was followed by anti-CD3/CD28 stimulation, and the supernatants and cells were harvested at 16 h for analyses.

### Measurement of CCL4 by ELISA

Supernatants from primary naïve CD8 T cells were collected at 16 and 48 h after anti-CD3/CD28 stimulation and from the stimulated miR-125b-enriched naïve CD8 T cells that were collected at 16 h. The amount of CCL4 protein was determined by an ELISA kit (R&D Systems) according to the manufacturer's instruction. Concentrations were calculated according to the standard and normalized to the seeded number of cells among different samples.

### Establishment of miR-125-overexpressing Jurkat cells

The miR-125b-1 plasmid mentioned above was transfected into Jurkat cells using Cell Line Nucleofector Kit V (Lonza) following the manufacturer's instructions and selected under 200 ng/mL puromycin. The transfected cells were maintained under 100 ng/mL puromycin in RPMI 1640 with 10% FBS and penicillin (10 U/mL)/streptomycin (10 μg/mL).

### Construction of wild-type and mutant 3′UTR of CCL4

The 3′UTR of the CCL4 gene containing a putative miR-125b binding site was amplified from human genomic DNA. The primers were CCL4-3′UTR: forward (5′-tttctcgagCCAAAAGAAGCAAGC-3′) and backward (5′-ttttgcggccgcGCAACAGCAGAGAAAC-3′). The amplified PCR fragment was digested with *XhoI* plus *NotI* and cloned into the pSiCheck-2 dual luciferase reporter (Promega, Madison, WI, USA). The ‘seed’ deleted 3′UTR of CCL4 lacking of 7-nt of the binding site was generated from the WT reporter plasmid with primers (forward: 5′-tttctcgagCCAAAAGAAGCAAGC-3′ and backward: 5′-gagtggtgaccTAGACTTCCTGTCTCTGAGCAGC-3′). The amplified PCR fragment was digested with *XhoI* plus *BstEII* and inserted into the corresponding site of the WT 3′UTR of CCL4 reporter plasmid.

### Luciferase reporter assay

The assay for luciferase activity was performed according to the manufacturer's instructions. Briefly, 2 μg pSiCheck-2 containing WT or ‘seed’ deleted 3′UTR of CCL4 reporter plasmid was transfected into miR-125b enriched Jurkat cells. Luciferase activities were assayed 36 h after transfection using the Dual-Luciferase Reporter Assay System (Promega) on GloMax®-Multi Detection System (Promega).

### Statistical analysis

Data are expressed as mean ± SEM, and significance was assessed using the Student's t-test as *P *<* *0.05. To address multiple comparisons, we have adjusted the *P*-values in our comparisons with Benjamini–Hochberg procedure. Adjusted *P*-value (q-value) < 0.05 was considered significant as indicated in the text and figures. To account for donor variation, a multifactor ANOVA was performed in R with age group, cell type, and donor identity as the three independent factors. The donor identity factor was not significant (*P *=* *0.88), nor was any interaction between donor identity and age group, or donor identity with cell-type significant. Linear regression analysis (R^2^) was used in Function from Excel.

## Supporting Information

**Fig. S1** Expression of CCL4 mRNA and miR-125b in memory CD8 T cells, naïve and memory CD4 T cells, and B cells. **Fig. S2** An inverse correlation of CCL4 mRNA and miR-125b expression in human immune cells.

**Table S1** Information of the study subjects and their use in the experiments.
