# Supplementary figures and images for: MicroRNA-125b modulates inflammatory chemokine CCL4 expression in immune cells and its reduction causes CCL4 increase with age
Source: Aging Cell. 2015 Jan 23;14(2):200–8. doi: 10.1111/acel.12294 (PMC4364832; doi:10.1111/acel.12294)

Fig. S1

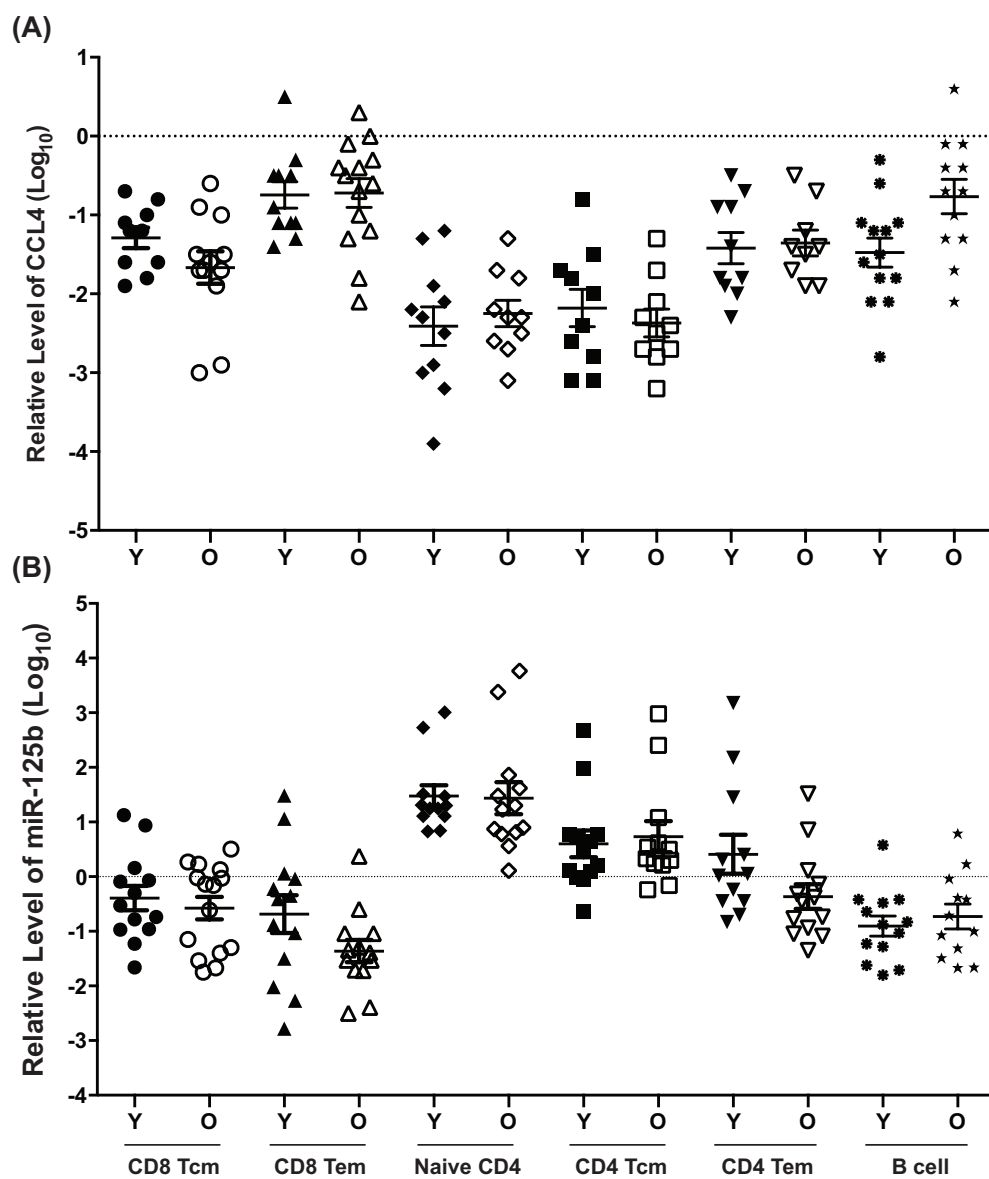

Fig. S2

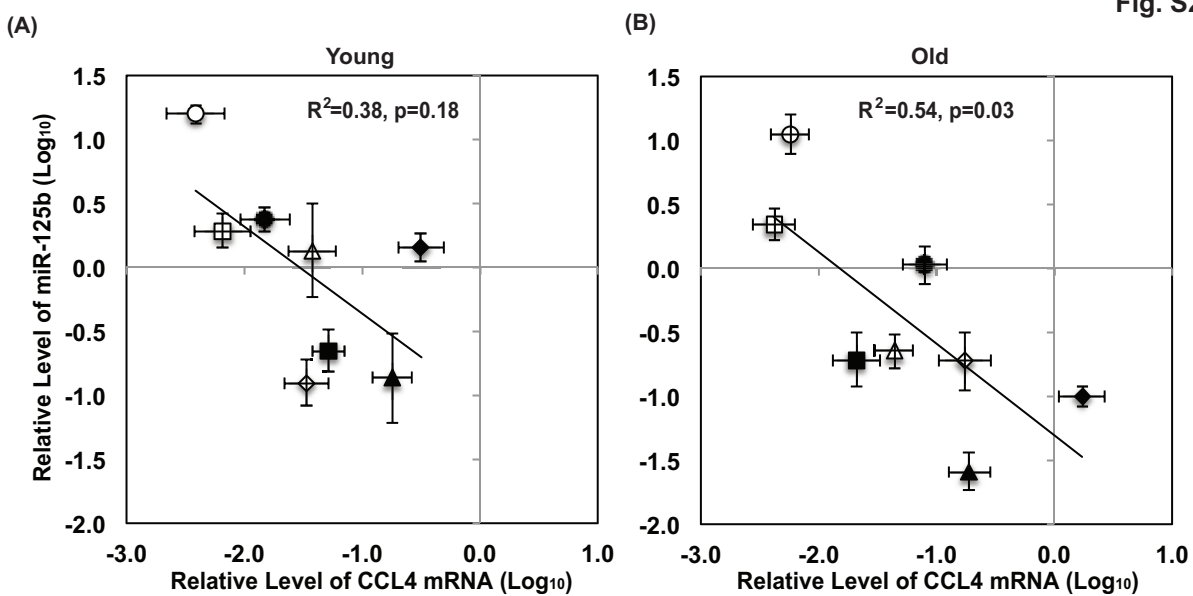

Supplement: Supplementary file 1 [file acel0014-0200-sd1.pdf]
