# Supplementary material for: MicroRNA-125b modulates inflammatory chemokine CCL4 expression in immune cells and its reduction causes CCL4 increase with age
Source: Aging Cell. 2015 Jan 23;14(2):200–8. doi: 10.1111/acel.12294 (PMC4364832; doi:10.1111/acel.12294)
Supplement: Supplementary file 2 [file acel0014-0200-sd2.pdf]

Table S1 Information of the study subjects and their use in the experiments

Subject #	Age	Gender	Monocyte	CD8			CD4			B cell	Time course	Transfection	Monocyte surface staining
				Naïve	CM	EM	Naïve	CM	EM				
1	21	M	Yes	No	No	No	Yes	Yes	No	Yes	No	No	No
2	21	M	Yes	No	No	No	No	No	No	Yes	No	No	No
3	23	M	No	Yes	Yes	Yes	No	No	No	No	Yes	Yes	No
4	23	M	Yes	No	No	No	No	No	No	No	No	No	No
5	24	F	No	Yes	No	No	Yes	No	No	No	Yes	No	No
6	24	F	No	No	No	No	No	No	No	No	No	No	Yes
7	24	M	Yes	Yes	Yes	Yes	Yes	Yes	Yes	Yes	Yes	No	No
8	24	M	No	No	No	No	No	No	No	No	Yes	No	Yes
9	25	M	Yes	No	No	No	No	No	No	Yes	No	No	No
10	26	F	No	Yes	No	Yes	Yes	Yes	Yes	No	Yes	Yes	No
11	26	F	Yes	Yes	No	No	Yes	No	No	No	Yes	No	No
12	26	M	Yes	Yes	Yes	Yes	No	No	No	Yes	Yes	No	No
13	26	M	Yes	No	No	No	No	No	No	Yes	No	No	No
14	27	F	No	No	No	No	No	No	No	No	No	Yes	No
15	27	M	Yes	Yes	No	Yes	Yes	Yes	No	Yes	Yes	Yes	No
16	28	M	No	No	No	No	No	No	No	No	No	Yes	No
17	28	M	Yes	Yes	Yes	Yes	Yes	Yes	Yes	Yes	Yes	No	No
18	28	M	Yes	No	No	No	No	No	No	Yes	No	No	No
19	29	F	No	Yes	Yes	Yes	No	No	No	No	Yes	No	No
20	29	M	Yes	Yes	Yes	Yes	Yes	Yes	Yes	Yes	Yes	No	No
21	29	M	No	Yes	Yes	Yes	No	Yes	Yes	No	Yes	Yes	No
22	29	M	No	Yes	No	No	Yes	Yes	Yes	No	Yes	No	No
23	30	M	No	Yes	No	No	Yes	No	No	No	Yes	No	No
24	30	M	No	No	No	No	No	No	No	No	No	Yes	Yes
25	31	F	Yes	Yes	Yes	No	No	No	No	Yes	Yes	Yes	No
26	31	F	Yes	No	No	No	No	No	No	No	No	Yes	Yes
27	31	M	No	Yes	Yes	No	Yes	Yes	Yes	No	Yes	Yes	No
28	31	M	Yes	No	No	No	No	No	No	No	No	Yes	Yes
29	32	M	No	Yes	No	No	Yes	Yes	Yes	No	Yes	No	No
30	33	M	Yes	Yes	No	No	Yes	Yes	No	Yes	Yes	Yes	No
31	34	F	No	Yes	No	No	Yes	No	Yes	No	No	No	No
32	34	M	Yes	Yes	Yes	Yes	No	No	Yes	Yes	Yes	Yes	Yes
33	34	M	No	No	No	No	No	No	No	No	Yes	Yes	No
34	35	M	No	No	No	No	No	No	No	No	No	Yes	No
35	36	F	Yes	No	No	No	No	No	No	No	No	Yes	Yes
36	37	M	Yes	No	Yes	Yes	No	No	No	No	Yes	No	No
37	37	M	Yes	Yes	Yes	Yes	No	No	No	Yes	Yes	No	No
38	37	M	No	No	No	No	No	No	No	No	No	Yes	No
39	38	M	No	No	No	No	No	No	No	No	No	No	Yes
40	39	F	No	No	No	No	No	No	No	No	No	No	Yes
41	39	F	No	No	No	No	No	No	No	No	No	No	Yes
42	39	F	No	No	No	No	No	No	No	No	No	No	Yes
43	39	M	No	No	No	No	No	No	No	No	No	No	Yes
44	41	F	No	No	No	No	No	No	No	No	Yes	No	Yes
45	41	F	No	No	No	No	No	No	No	No	No	No	Yes
46	41	M	No	No	No	No	No	No	No	No	No	Yes	No
47	44	M	No	No	No	No	No	No	No	No	No	Yes	No

48	45	M	No	No	No	No	No	No	No	No	No	Yes	No
49	46	M	No	No	No	No	No	No	No	No	No	Yes	No
50	47	M	No	No	No	No	No	No	No	No	No	Yes	No
51	50	M	No	No	No	No	No	No	No	No	No	Yes	No
52	52	M	No	No	No	No	No	No	No	No	No	Yes	No
53	54	M	No	No	No	No	No	No	No	No	No	Yes	No
54	55	M	No	No	No	No	No	No	No	No	No	Yes	No
55	60	M	No	No	No	No	No	No	No	No	No	Yes	No
56	60	M	No	No	No	No	No	No	No	No	Yes	Yes	No
57	65	M	No	No	No	No	No	No	No	No	No	Yes	No
58	67	M	No	No	No	No	No	No	No	No	No	Yes	No
59	69	M	No	No	No	No	No	No	No	No	No	No	Yes
60	70	F	Yes	Yes	Yes	Yes	Yes	Yes	Yes	Yes	Yes	No	No
61	70	F	No	Yes	Yes	No	Yes	No	No	No	Yes	No	No
62	70	M	No	Yes	No	No	No	No	No	No	No	No	No
63	71	F	No	Yes	Yes	No	Yes	No	No	No	Yes	No	No
64	71	F	Yes	Yes	Yes	No	No	No	No	Yes	No	No	No
65	71	F	No	Yes	No	No	Yes	Yes	Yes	No	Yes	No	No
66	71	F	Yes	No	No	Yes	Yes	No	Yes	Yes	No	No	No
67	71	M	No	Yes	No	No	No	Yes	No	No	Yes	No	No
68	71	M	No	No	No	No	No	No	No	No	No	Yes	No
69	71	M	No	Yes	No	Yes	No	No	No	No	No	No	No
70	71	M	Yes	No	No	No	No	No	No	No	No	No	No
71	72	F	No	No	No	No	No	No	No	No	No	No	Yes
72	72	F	No	Yes	Yes	Yes	No	No	No	No	Yes	No	No
73	72	M	No	Yes	Yes	Yes	Yes	Yes	Yes	No	Yes	No	No
74	72	M	Yes	No	No	No	No	No	No	No	No	No	No
75	73	M	No	Yes	Yes	Yes	No	No	No	No	Yes	No	No
76	73	M	No	No	No	No	No	Yes	No	No	No	No	No
77	74	F	No	Yes	Yes	Yes	No	No	No	No	Yes	No	No
78	74	F	Yes	No	No	No	No	No	No	Yes	No	No	No
79	74	M	Yes	Yes	No	Yes	No	No	Yes	Yes	No	No	No
80	75	F	No	Yes	Yes	No	Yes	Yes	Yes	No	Yes	No	No
81	75	F	No	No	No	No	No	No	No	Yes	No	No	No
82	75	M	No	Yes	Yes	Yes	No	No	No	No	Yes	No	No
83	75	M	No	No	No	No	No	No	No	No	No	No	Yes
84	75	M	No	No	No	No	No	No	No	No	No	No	Yes
85	75	M	Yes	No	No	Yes	No	Yes	Yes	Yes	No	No	No
86	75	M	No	Yes	No	No	No	No	No	No	Yes	No	No
87	76	F	No	Yes	Yes	Yes	No	No	No	No	Yes	No	No
88	76	M	Yes	Yes	No	Yes	No	No	Yes	Yes	No	No	No
89	77	M	No	No	No	No	No	No	No	No	No	No	Yes
90	78	M	No	Yes	No	No	No	No	No	No	Yes	No	No
91	78	M	No	Yes	Yes	Yes	No	No	No	No	Yes	No	No
92	78	M	Yes	No	No	No	No	No	No	No	No	No	No
93	79	F	Yes	No	No	No	No	No	No	No	No	No	No
94	79	M	Yes	No	No	No	No	No	No	No	No	No	No
95	80	M	No	Yes	No	No	Yes	No	Yes	No	Yes	No	No
96	81	F	Yes	No	Yes	Yes	Yes	Yes	Yes	Yes	Yes	No	No
97	81	M	Yes	No	No	No	No	No	No	No	No	No	No
98	82	F	No	No	No	No	No	No	No	No	No	No	Yes

99	82	M	No	Yes	Yes	No	Yes	Yes	No	No	Yes	No	No
100	82	M	No	No	No	No	No	No	No	No	Yes	No	Yes
101	84	M	No	No	No	No	No	No	No	No	No	No	Yes
102	85	M	No	No	No	No	No	No	No	No	No	No	Yes
103	85	M	Yes	Yes	Yes	Yes	Yes	Yes	Yes	Yes	Yes	No	No
104	86	F	No	No	No	No	No	No	No	Yes	No	No	No
105	89	F	Yes	No	No	No	No	No	No	No	No	No	No
106	89	M	Yes	No	No	No	No	No	No	No	No	No	No
Total			37	43	27	28	25	21	21	25	44	30	23

Subjects listed under 8 types of immune cells (columns 4-11) were used for the quantitative RT-PCR of CCL4, miR-125b, and pri-miR-125b 1&2 (Fig. 1 and S1)

Subjects under the time course were used for stimulation of monocytes and naïve CD8 T cells in vitro with LPS and anti-CD3/38 respectively (Fig. 2 c & d, Fig. 4).

Subjects under "Transfection" were used for miR-125b inhibition (Fig. 3) and overexpression (Fig. 5).

Subjects under "monocyte surface staining" were used for analysis of monocyte activation markers by flow cytometry (Fig. 2 a & b).

CM=central memory and EM=effector memory.
